# Pharmacophore-Assisted Covalent Docking Identifies a Potential Covalent Inhibitor for Drug-Resistant Genotype 3 Variants of Hepatitis C Viral NS3/4A Serine Protease

**DOI:** 10.3390/v16081250

**Published:** 2024-08-03

**Authors:** Kanzal Iman, Muhammad Usman Mirza, Fazila Sadia, Matheus Froeyen, John F. Trant, Safee Ullah Chaudhary

**Affiliations:** 1 Biomedical Informatics & Engineering Research Laboratory, Department of Life Sciences, Lahore University of Management Sciences, Lahore 36000, Pakistan; ikanzaliman@gmail.com (K.I.); 19140006@lums.edu.pk (F.S.); 2 Department of Chemistry & Biochemistry, University of Windsor, Windsor, ON N9B 3P4, Canada; j.trant@uwindsor.ca; 3 Department of Pharmaceutical and Pharmacological Sciences, Rega Institute for Medical Research, KU Leuven—University of Leuven, B-3000 Leuven, Belgium; mathy.froeyen@kuleuven.be

**Keywords:** Hepatitis C virus, MD simulations, covalent inhibitor, drug resistance, pharmacophore-based virtual screening

## Abstract

The emergence of drug-resistance-inducing mutations in Hepatitis C virus (HCV) coupled with genotypic heterogeneity has made targeting NS3/4A serine protease difficult. In this work, we investigated the mutagenic variations in the binding pocket of Genotype 3 (G3) HCV NS3/4A and evaluated ligands for efficacious inhibition. We report mutations at 14 positions within the ligand-binding residues of HCV NS3/4A, including H57R and S139P within the catalytic triad. We then modelled each mutational variant for pharmacophore-based virtual screening (PBVS) followed by covalent docking towards identifying a potential covalent inhibitor, i.e., cpd-217. The binding stability of cpd-217 was then supported by molecular dynamic simulation followed by MM/GBSA binding free energy calculation. The free energy decomposition analysis indicated that the resistant mutants alter the HCV NS3/4A–ligand interaction, resulting in unbalanced energy distribution within the binding site, leading to drug resistance. Cpd-217 was identified as interacting with all NS3/4A G3 variants with significant covalent docking scores. In conclusion, cpd-217 emerges as a potential inhibitor of HCV NS3/4A G3 variants that warrants further in vitro and in vivo studies. This study provides a theoretical foundation for drug design and development targeting HCV G3 NS3/4A.

## 1. Introduction

Hepatitis C virus (HCV) is an enveloped, single-stranded, positive-sense RNA virus of the Flaviviridae family [1]. HCV is the causative agent of liver Hepatitis C infection, which has an estimated global prevalence of 2.5% [2]. Chronic HCV infections often lead to liver cirrhosis, hepatocellular carcinomas, and liver failure [2,3,4,5]. HCV isolates exhibit vast genetic heterogeneity [6], based on which the viral variants have been grouped into seven genotypes (G1–G7) and various subtypes [2,7,8,9]. G1 and G3 together account for over 80% of global infections [10,11]. In particular, G1 has a high prevalence in Europe and North and South America [12] and accounts for 46% of the overall HCV infections [11]. Meanwhile, G3 is more prevalent in Australia and South Asia [12] and is causative of 30% of the global HCV burden [10,11]. Other HCV genotypes (G2–G7) account for the remaining HCV infections and generally remain highly local concerns [11,13].

The HCV genome is 9.6 kb in length, translating into a precursor polyprotein of ~3000 residues [14]. This polyprotein is then enzymatically processed to yield four structural (core, envelope glycoproteins E1 and E2, and an ion channel p7) and six non-structural (NS) proteins (NS2, NS3, NS4A, NS4B, NS5A, and NS5B) [15]. The structural proteins are processed by host peptidases, while the non-structural proteins are cleaved by viral proteases, NS2 and NS3 [16]. In particular, NS2 is an auto-protease that is responsible for intramolecular cleavage between NS2 and NS3 of HCV polyprotein [17,18]. In the case of NS3, the protein has an N-terminal serine protease domain alongside a C-terminal helicase domain with NTPase activity [19,20,21,22]. NS3, together with its cofactor NS4A, makes a heterodimeric NS3/4A complex that cleaves four scissile peptide bonds (3–4A, 4A–4B, 4B–5A, and 5A–5B) between non-structural proteins [23,24,25]. The multifunctional protein NS3 additionally hydrolyses two signaling proteins, including mitochondrial antiviral signaling protein (MAVS) and C-terminal Toll-interleukin-1 receptor (TIR) domain-containing adaptor inducing IFN-b (TRIF) [26]. Importantly, MAVS and TRIF are responsible for activating the immune response to viral infections [26,27]. NS3/4A-mediated processing of viral and host proteins is central to viral replication and innate immune response, rendering it an ideal therapeutic target [19,20,21,22,28,29].

To date, several NS3/4A-targeting direct-acting antivirals (DAAs), including boceprevir [30], telaprevir [31,32], narlaprevir [33], simeprevir, grazoprevir, paritaprevir, and glecaprevir, have been approved by the Food and Drug Administration (FDA) [24,25,34] that act to inhibit viral replication [15,35,36,37,38,39,40]. Three of these marketed drugs are covalent (serine-trap) inhibitors [34]. Nucleophilic attack by the hydroxyl group of Ser139 at the NS3 protease catalytic triad on the electrophilic warhead leads to the formation of a stable covalent bond between NS3/4A and the inhibitor [33,41,42,43,44]. The nature of the electrophile dictates if covalent inhibition is reversible or irreversible [45]. Covalent NS3/4A inhibitors have demonstrated a good safety profile in addition to being potent in their antiviral activity [46,47]. However, the genetic sequence heterogeneity within NS3/4A impacts the efficiency of NS3/4A inhibitors [24,25]. Consequently, the antiviral response varies significantly between HCV genotypes [48]. NS3/4A of HCV G1 exhibits the highest sustained virological response (SVR) rate (up to 99%) to third-generation NS3/4A inhibitors [19,49,50,51,52]. However, the efficacy of DAAs targeting NS3/4A has been shown to be lower in other genotypes [53,54,55,56,57,58]. Specifically, in G3, a predominant HCV genotype in South Asia, including Pakistan [59]—a low- to middle-income country—DAA targeting of NS3/4A has presented a major challenge towards affordable HCV treatment [60,61,62,63]. In addition to decreased SVR rates in response to DAAs [64], NS3/4A G3 is associated with an increased risk of liver cirrhosis and hepatocellular cancer [64].

Besides genotypic variation, polymorphism in NS3/4A is also associated with conferring resistance to approved DAAs [65,66]. These single-site mutations [67] within the ligand-binding pocket of NS3/4A G3 [67,68,69,70] cause resistance in drug binding [65,67,70], leading to the emergence of drug-resistant variants [2,9,71]. Nearly 50% of DAA treatment failures have reported resistance-associated substitutions (RASs) [72,73]. The frequently observed NS3/4A RASs include V36, T54, V55, Q80, R155, A156, and D168 [74]. Specifically, A156F/N/V, V36A+R155K/T, V36M+R155T, V36A/M+A156T, T54A+A156S, T54S+A156S/T, and V36M+T54S+R155K have reported resistance to telaprevir [75]. Therefore, the occurrence of RASs within NS3/4A has challenged the clinical success of approved DAAs [76]. Specifically, D168Q polymorphism has rendered NS3/4A G3 “naturally resistant” to most DAAs [66,68,77]. Moreover, the lack of availability of an NS3/4A G3 crystal structure [78,79] further limits the investigation of genotype-specific mutations [67,80] and their impact on NS3/4A’s interaction with inhibitors [81]. Taken together, NS3/4A G3 remains a difficult-to-treat strain.

In recent decades, in silico drug discovery has revolutionised conventional pharmaceutical development [82,83,84]. Various in silico methods have assisted in the discovery of potent antiviral compounds against viruses, such as dengue [85], zika [86,87], influenza [88], SARS-CoV-1 [89,90], and Ebola [91]. Following this, we have combined in silico techniques [90,92,93,94] to identify small molecules targeting HCV NS3/4A G3. In this work, we have modelled NS3/4A variants by considering G3-specific mutations at fourteen positions within the ligand-binding pocket, including residues of the catalytic triad (F43L, H57R, Q80K, R123T, I132L, Y134C/R, S139P, R155G, A156T, V158A, C159V, D168Q, C525W/Y, and Q526H/R). This was followed by pharmacophore-based virtual screening (PBVS) and a covalent docking protocol to identify potential covalent inhibitors. Binding interactions of potential antivirals identified in this study were validated using molecular dynamics (MD) simulations. This study reports a potential anti-HCV compound targeting NS3/4A G3-specific mutations exhibiting strong binding affinity scores that has the potential to be directly tested for in vitro and in vivo studies for HCV drug development.

## 2. Materials and Methods

### 2.1. Sequence Retrieval and Modelling HCV NS3/4A G3

In silico modelling was initiated by retrieving sequences of HCV NS3/4A Genotype 3 (G3) from the National Center for Biotechnology Information (NCBI) protein database [95] (see Supplementary Table S1). Inhibitor-bound HCV NS3/4A complexes were retrieved from the Protein Data Bank (PDB) [96] for binding site analysis (see Supplementary Table S1). The binding pocket of HCV NS3/4A was investigated using LigPlot+ v.2.1 [97] and Maestro v.11 (Schrödinger, LLC, New York, NY, USA) [98], and the ligand-binding residues were identified (see Supplementary Table S2). Multiple sequence alignment (MSA) was performed using Clustal Omega [99], and the identified interacting residues identified were analysed for mutations within G3-specific sequences retrieved from the NCBI protein database [95] (see Supplementary Table S2). A representative sequence for each mutation was selected as a G3-specific variant for modelling (see Supplementary Table S3). ESPript 3.0 was used for MSA visualisation [100]. Templates for homology modelling were identified from PDB using the Protein Basic Local Alignment Search Tool (BLASTP) algorithm [101] (see Supplementary Table S4). The co-crystallised inhibitor-bound HCV NS3/4A complex (PDB ID: 4A92) was selected as a template. Homology modelling of HCV NS3/4A G3-specific variants was performed using the SWISS-MODEL Server [102], an automated homology modelling resource.

### 2.2. 3D Structure Refinement and Validation

The predicted homology models were refined using GalaxyRefine [103]. Quality assessment of the models was performed by using FATCAT [104], MATRAS [105], ERRAT [106], ProSA [107], the SAVES Server [108], MolProbity [109], Verify 3D [110], PROCHECK [111], and Maestro [98]. Models with the least number of residues in the disallowed region were selected. The root mean square deviation (RMSD) values were calculated using CHIMERA [112] (see Supplementary Table S5).

### 2.3. Ligand-Based Pharmacophore Modelling and Virtual Screening

**Figure 1:**
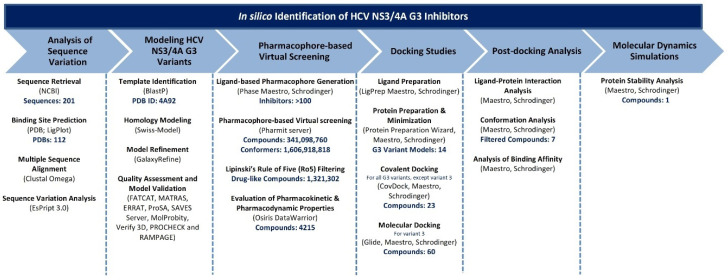
Framework for in silico analysis of HCV NS3/4A G3. Flowchart summarising the computational framework for modelling and targeting HCV NS3/4A G3 towards HCV treatment.

Ligand-based pharmacophore modelling was carried out with the Phase module of Maestro [113,114] against HCV NS3/4A. A set of over 100 HCV NS3/4A covalent and non-covalent inhibitors, including marketed drugs reported in the literature, were retrieved from the Protein Data Bank (PDB) (see Supplementary Table S6). The features of these ligands were extracted to generate the ligand-based pharmacophore hypotheses. The compounds were screened from small molecule databases, including ZINC [115], MolPort [116], PubChem [117], Mcule [118], and ChEMBL [119]. The screening was performed using the Pharmit web server [120], which uses a pharmacophore and molecular shape search. The resulting hits were refined by applying a series of filters, including Lipinski’s rule (RO5), with at least 2 violations [121]. The oral bioavailability assessment was performed using Osiris DataWarrior software v5.5.0 [122], and pharmacokinetic and pharmacodynamic properties of the screened ligands were evaluated. These strict criteria removed a huge dataset, and the remaining compounds were utilised for more in-depth covalent docking using Schrödinger’s Covalent Docking (CovDock) tool implemented in Maestro. The detailed framework applied in the study for targeting HCV NS3/4A G3 variants using in silico approaches is represented in Figure 1.

### 2.4. Preparation of HCV NS3/4A and Covalent Docking Protocol

The Protein Preparation Wizard in Maestro [123] was used to prepare modelled HCV NS3/4A G3 variants (G3.v1–G3.v14). The protein structure was pre-processed by assigning bond orders and adding missing hydrogen atoms using Epik [124] at pH 7.0 ± 2.0. Different tautomeric states were generated for each ligand. Moreover, two alternative stereoisomers were generated per ligand while retaining the specified chiralities. Next, the H-bond network was optimised with PROPKA [125] at pH 7.0. The histidine residues within the active site of serine protease were defined, including the histidine of the catalytic triad (His57). Moreover, all water molecules were removed, and a restrained minimisation was carried out using the newly optimised OPLS3e force field [98], and the convergence criterion of 0.3 Å RMSD was set for all heavy atoms. The compounds resulting from the ligand-based pharmacophore search were processed with the LigPrep module of Maestro [126].

Because the active site of HCV NS3/4A contains a catalytic serine (Ser139), it is possible to target it with compounds that bind covalently. For example, within the crystal structure of HCV NS3/4A protease (PDB ID: 3LON), a ketoamide inhibitor narlaprevir is covalently attached to Ser139 of the catalytic triad. Therefore, we carried out a covalent docking protocol to screen the resultant compounds using Schrödinger’s Covalent Docking (CovDock) tool [127] implemented in the Maestro molecular modelling package [98]. In addition to the Michael addition reaction, we also explored other reaction types, including nucleophilic addition to double bond or triple bond.

The centre of the covalent docking site was defined as the centroid of the catalytic Ser139 residue, and a cube grid centred on this point was set with an edge length of ≤20 Å. The compounds were initially docked using the fast-virtual screening mode of CovDock in Maestro and the Michael addition reaction to investigate the formation of covalent interaction with Ser139. The compounds that successfully interacted covalently with Ser139 were again docked using a thorough pose prediction mode in Maestro [98], and a maximum number of 100 poses was selected. The cut-off at 2.5 kcal/mol was set to retain poses for further refinement. The poses were ranked using a Prime [128] MMGBSA score that employs a variable dielectric generalised Born solvation model (VSGB 2.1) [129,130] in the OPLS3e force field [131]. These steps were repeated for other reaction types, including nucleophilic addition to a double and a triple bond, nucleophilic substitution, and aryl and nitrile activated conjugate addition to the alkyne. The top-ranking compounds were selected for validation using MD Simulations. 

For G3.v3 with S139P mutation, molecular (non-covalent) docking-based virtual screening was performed to identify potential protease inhibitors. The variant was prepared using a protein preparation wizard in Maestro [98]. Hydrogens were added, and proper bond order and energy minimisation were performed. The molecular interaction grid was centred at the active site containing the catalytic triad and prepared using the receptor grid generation module in Maestro [98]. Molecular docking was performed on selected compounds from pharmacophore-based virtual screening using the Glide program of Schrodinger [132]. The resulting lead compounds were analysed for the binding interaction with the catalytic triad.

### 2.5. Molecular Dynamics Simulations

Molecular dynamics (MD) simulations of each complex were performed to investigate the binding potential of the hit compounds interacting with the residues inside of the active site of HCV NS3/4A G3. MD simulations were performed for 100 ns using AMBER 20 simulation package [133]. Simulation trajectories elucidated complex stability, and interaction profiles were investigated. An MD simulation protocol as described previously [134,135,136] was implemented; however, the length of the production run was increased from 20 to 100 ns. Parameterisation was performed, and a new molecular topology file for each ligand was created. This was initiated from each ligand and ended in covalently bound Ser139. The topology and coordinate files of the complexes were generated using the tleap program of AMBER. The Antechamber package of AmberTools was utilised, and parameters were extracted from the GAFF force field (GAFF) [137]. Counter ions were added around the ligand–protease complex to neutralise the charges of each simulation system. The complex was centred in a dodecahedral TIP3P [138] water box with a distance of 10 Å between the solute and the box edge. To maintain a constant bond length, covalent bonds were constrained using the SHAKE algorithm [139]. The system was heated and equilibrated after a stepwise minimisation. A production run was performed at 300 K and 1 bar pressure for a period of 100 ns. The time step of 2 fs was set, and the trajectory snapshots were saved every 2 ps for onward analysis using the CPPTRAJ program [140] of AMBER.

### 2.6. Binding Free Energy Calculations

The binding free energies (ΔGbind) of HCV NS3/4A G3 variants complexed with the most promising hit compounds were calculated using the MM-GBSA method, implemented in AMBER 20. For each system, 10000 snapshots were generated from the last 50 ns stable trajectories with an interval of 5 ps. The total binding free energy was calculated as a sum of solvation free energy (ΔGsol) and the molecular mechanics binding energy (ΔEMM), as given below:

ΔEMM = ΔEint + ΔEele + ΔEvdw (1)

ΔGsol = ΔGpl + ΔGnp (2)

ΔGtotal = ΔEMM + ΔGsol(3)

ΔG_bind_ = ΔE_MM_ + ΔG_sol_ − TΔS (4)

Here, ΔEMM includes electrostatic energy (ΔEele), internal energy (ΔEint), van der Waals energy (ΔEvdw), and the polar (ΔGp) and non-polar (ΔGnp) energy components contributing to the total solvation free energy (ΔGsol). ΔGtotal is the free energy of binding evaluated for both MM-GBSA and MM-PBSA methods after entropic calculations (-TΔS). Per-residue energy decomposition analysis was performed using the MM-GBSA method to estimate the contribution of interacting residues towards ligand binding. The binding energy was calculated as ΔGresidue using the equation below:

ΔGresidue = ΔEMM + ΔGsol (5)

Here, the ΔGresidue denotes the total energy obtained from sidechain and backbone energy decomposition. Only those amino acids were considered that were within 8 Å of the active site. The sum of energy contributions from each residue is equal to the system’s overall binding energy [141]. MM-GBSA is a widely used method for estimating the free energy of binding of small ligands to biological macromolecules. It relies on molecular dynamics simulations of the receptor–ligand complex and offers more reliable estimates than empirical scoring functions. This strategy is well-established for protein–ligand and protein–protein interaction studies and provides valuable insights into binding affinities and interaction mechanisms [94,142].

## 3. Results and Discussion

### 3.1. Analysis of Sequence Variation and Molecular Modelling

**Figure 2:**
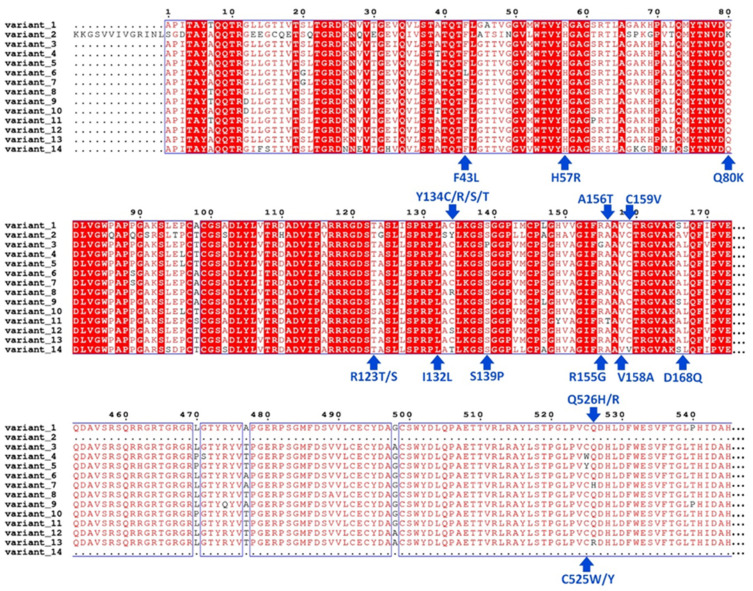
Multiple sequence alignment of HCV NS3/4A Genotype 3 variants. Amino acid substitutions at 14 positions are identified with the arrow in blue. The respective mutations at specified positions are identified in blue. The conserved residues are highlighted in red. Note: Variant_1 to variant_14 as G3.v1 to G3.v14.

The results from the sequence alignment revealed the catalytic triad of HCV NS3/4A G3 as a site of viral polymorphism due to mutations, including H57R and S139P (Figure 2). The analysis further showed mutations within HCV NS3/4A ligand-interacting residues at positions 43, 80, 123, 132, 134, 139, 155, 156, 158, 159, 168, 525, and 526. Of these residues, mutations at positions 80 [143,144,145], 155 [67,146], 156 [147], 123 [65,66], and 168 [148,149,150,151] have been frequently reported as drug-resistance-associated substitutions (RASs) in HCV NS3/4A. A representative sequence for each mutation was selected to model the HCV NS3/4A G3 variant (see Supplementary Table S3). 

The crystal structure of HCV NS3/4A protease-helicase G1b (PDB ID: 4A92) with the sequence identity in the range 74–82% (E-value 0.0) for 15 HCV NS3/4A G3 variants was selected as a template for homology modelling (see Supplementary Table S4). The structure was minimised and used for the HCV3/4A modelling (see Supplementary Figure S1). 

The predicted structure quality score was computed using ERRAT [106] and Verify 3D [110] and ranged between 91.33 and 94.85. The MolProbity [109] quality values ranged between 1.50 and 1.61. The superimposition of variants with the template indicated root mean square deviation (RMSD) values in the range of 0.28 Å–0.30 Å (see Supplementary Table S5). The Ramachandran plots generated for each variant model showed model residues >95% in the favoured regions (see Supplementary Figure S2). Furthermore, we evaluated the reliability of the HCV NS3/4A G3 wildtype (WT) model with its respective templates (PDB IDs: 4B75, 1CU1, 3O8B, 5WDX and 4B6E), and root mean square fluctuations (RMSFs) were compared after 100 ns MD simulations. The results indicated no significant fluctuations, and the movements of secondary structural elements were analogous to the templates, while the catalytic triad remained converged below 1Å (see Supplementary Figure S1).

### 3.2. Ligand-Based Pharmacophore Search

Next, we incorporated both covalent and non-covalent features of reported HCV inhibitors to build a pharmacophore hypothesis against HCV NS3/4A G3. To identify compounds with the most likely interacting substructures and the potential warheads that can establish covalent bonds with catalytic Ser139, a set of well-defined HCV NS3/4A inhibitors was retrieved (see Supplementary Table S6). Using the PHASE 4.0 module of Schrodinger molecular modelling [98], pharmacophore hypotheses were generated. Five hypotheses were generated, and the hypothesis with the best survival score (5.080) and fitness score was selected. This model consisted of four features (AAHH), including two hydrogen bond acceptors (A4 and A6) and two hydrophobic groups (H14 and H15). The pharmacophore hypothesis was validated with receiver operating characteristic (ROC) analysis [152] to assess its ability to correctly classify compounds as active or inactive [153]. The performance of the pharmacophore hypothesis was further evaluated according to the area under the curve (AUC) of the corresponding ROC curve (see Supplementary Figure S3). The validated pharmacophore model’s AAHH curve (see Supplementary Figure S4) was used to screen databases in the Pharmit web server [120] containing 341,098,760 compounds with 1,606,918,818 conformations. Based upon Lipinski’s rule of five (Ro5), 1,321,302 drug-like compounds were obtained, which were further filtered based on ADMET (absorption, distribution, metabolism, excretion, and toxicity) properties in Osiris DataWarrior software [122]. Unique compounds from the filtered 4215 compounds were prepared for docking studies (see Supplementary Table S7).

### 3.3. Covalent Docking-Based Virtual Screening 

Ser139 is a key residue in the active site of HCV NS3/4A, which makes it an attractive target for the design of covalent inhibitors [33,154,155]. Covalent docking-based virtual screening was performed to target the reactive nucleophilic Ser139 to identify compounds with reactive electrophilic moieties. The virtual screening was conducted in two steps, involving (i) the selection of candidate ligands based on their relevant conformation such that reactive groups are in close proximity to Ser139, and (ii) a virtual chemical reaction between the reactive groups, leading to the formation of a stable covalent bond (S-C) [156,157]. Ligand poses within a 5 Å distance cut-off from Ser139 were kept, forming a covalent bond (S-C) according to the reaction type. To identify potential covalent inhibitors from a pharmacophore-based screened library containing chemical warheads, the covalent binding reactions used included the Michael addition, nucleophilic addition to a double and a triple bond, nucleophilic substitution, and aryl and nitrile activated conjugate addition to alkyne. The free energies of binding were calculated using the Prime/MM-GBSA method for all of the docked poses (see Section 2 for details). The compounds with the lowest Prime/MM-GBSA and/or CovDock scores were considered for further molecular inspection.

**Figure 3:**
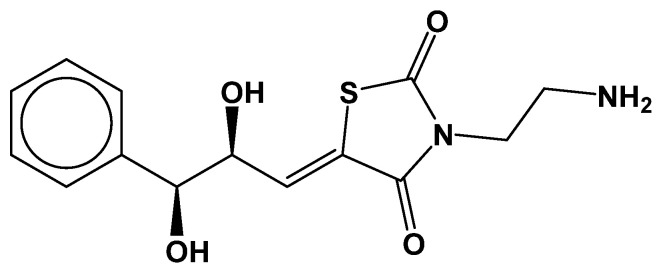
Chemical structure of CHEMBL569970 (cpd-217; PubChem45485999). The lead indicated binding potential with all HCV NS3/4A variants.

Overall, a total of 23 compounds from the results of the Michael addition screening were identified, while no compounds could be identified from other reaction types: nucleophilic addition to double bond and nitrile activated conjugate addition to alkyne. Inspection of the structures and binding poses led to the selection of seven compounds (see Supplementary Table S8 and Supplementary Figure S5). Amongst these, only one inhibitor candidate, CHEMBL569970 (cpd-217: PubChem45485999) (Figure 3), reported a high CovDock docking score with all variants incorporating G3-specific mutations, and it was selected for a detailed interaction analysis. A detailed interaction analysis of cpd-217, and its stability inside of the active site of HCV NS3/4A G3 variants, was assessed through 50 ns MD simulations, and binding free energies were calculated.

Molecular docking of G3.v1 resulted in 60 compounds (see Supplementary Table S9 and Supplementary Figure S6). Ligand interaction analysis was performed to filter leads. The compound cpd-217 indicated stable interactions, which were validated through MD simulation analysis.

### 3.4. Molecular Insights of Identified Potential Covalent Inhibitor (cpd-217)

**Figure 4:**
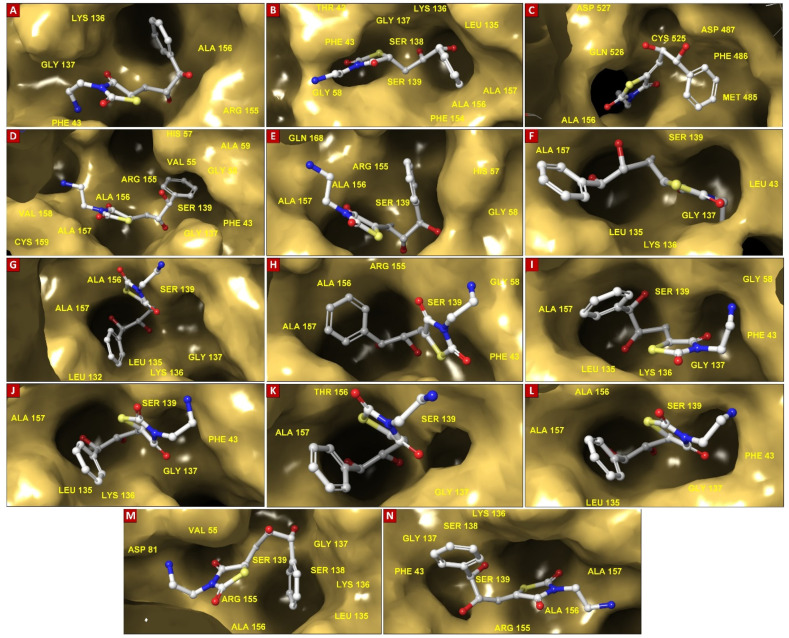
Surface representation of the binding pocket of HCV NS3/4A G3 variants docked with CHEMBL569970 (cps-217; PubChem45485999). Key interacting residues including Thr42, Leu/Phe43, Val55, Gly58, Asp81, Leu135, Lys136, Gly137, Ser138, Ser139, Phe154, Arg155, Ala156, Ala157, Met485, Phe486, Gly525, Gln526, Asp527, and Asp528 are labelled. All HCV NS3/4A G3 variants 1 to 14 are highlighted in (**A**–**N**), respectively.

The docked complexes of a potential hit with HCV NS3/4A variants were investigated for molecular interactions governed by covalent bond formation. The structures of the lead compound and their molecular interactions with the binding pocket of HCV NS3/4A G3 variants, v1 to v14 (Figure 4A–N), along with the corresponding reaction sites between the chemical warhead of the lead compound and the catalytic Ser139 are displayed in Figure 4. 

Overall, among cpd-217/G3 complexes, the catalytic serine (Ser139) was covalently bound to the Michael acceptor warhead (≤1.9 Å distance), except HCV NS3/4A G3.v3, which had proline instead of serine at position 139. The residues within the binding pocket [158] of all variants interacting with cpd-217 were compared with the residues interacting with the alpha-ketoamide inhibitor, boceprevir (PDB: 2O8) (see Supplementary Figure S7). Like boceprevir, cpd-217 formed interactions with Gln41, His57, Ile132, Leu135, Lys136, Gly137, Ser138, Ser139, Phe154, Arg155, Ala156, Ala157, and Val158. Additionally, cpd-217 interacted with Thr42, Phe43, Val55, Gly58, Asp81, Val158, Cys159, Met485, Phe486, Asp487, Ser488, Val524, Cys525, Gln526, and His528 (Table 1). All variants exhibited R123T (except R123S in G3.v10), I132L, Y134C (except G3.v2), and D168Q mutations, of which R123T and D168Q are frequently reported as RASs. Additionally, in G3.v1 with the H57R mutation, cpd-217 made five H-bonds with Gln41, Thr42, mutated catalytic His at position 57 to Arg, Leu135, and Arg155. In G3.v2 with the Q80K mutation, cpd-217 made four H-bonds with Gln41, His57, Leu135, and Gly137. G3.v3 exhibited catalytic Ser139 mutation to Pro, in addition to R155G mutation. Cpd-217 made three H-bonds with Asp81, Asp487, and Gln526 and established a salt–bridge interaction with Asp81. In G3.v4 (C525W) and G3.v5 (C525Y), cpd-217 made four H-bonds with Gly137, Ser139, and Ala157. Additionally, a π–π interaction was observed with His57 in G3.v4. In G3.v6 with the F43L mutation, cpd-217 made three H-bonds with residues Thr42, Gly137, and Ala157. Cpd-217 was observed making two H-bonds with Leu137 and Gly137 in G3.v7 (Q526H) and an additional H-bond with His57 in G3.v8 (Y134R). In G3.v9 (V158A), cpd-217 made π–cation interaction with Lys136 in addition to interacting with Gln41, His57, and Arg155, making three H-bonds. Cpd-217 made four H-bonds with His57, Leu135, and Ser139 in addition to covalently inhibiting Ser139 in G3.v10 with the R123S mutation. In G3.v11 with the A156T mutation, no H-bond formation was observed. However, cpd-217 made six H-bonds with Gln41, His57, Leu135, and Ser139 in G3.v12 exhibiting the Y134S mutation. A salt–bridge interaction between cpd-217 and Asp81 was observed in G3.v13 with the Q526R mutation. Additionally, two H-bonds between cpd-217 and Asp81 and Gly137 were observed. Cpd-217 made five H-bonds with Gln41, Gly137, Ser139, and Ala157 in G3.v14 with Y134T and C159V mutations.

These H-bonds were evinced from the reported crystal complexes, where eight hydrogen bonds are conserved between protease residues (Gly137, Ser138, Ser139, Arg155, Ala157, Ser159) and the viral substrate residues [159], indicating that they played a vital role in binding within wildtype (WT) systems [34].

**Figure 5:**
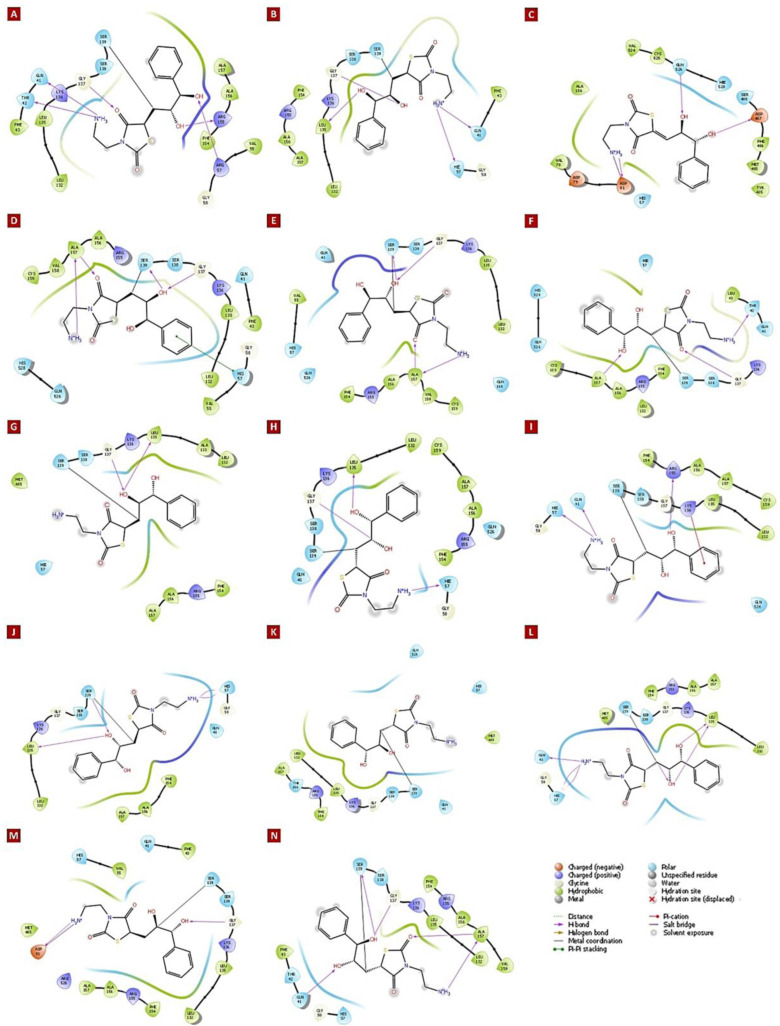
Ligand interaction diagrams of CHEMBL569970 (PubChem45485999) with residues inside of the binding pocket of HCV NS3/4A G3 variants. Residues making hydrogen bond (H-bond) interactions include Gln41, Thr42, His/Arg57, Asp81, Leu135, Gly137, Ser139, Arg155, Ala157, Asp487, and Gln526. Asp81 is involved in making the salt bridge in G3.v3 and 13. Lys136 (G3.v10) and H57 (G3.v4) are involved in hydrophobic interactions, including Pi–cation and Pi–Pi stacking, respectively. All HCV NS3/4A G3 variants 1 to 14 are highlighted in (**A**–**N**), respectively.

We observed that mutations within the catalytic triad residues His57Arg and Ser139Pro hindered interaction of cpd-217 with Gly137, a residue of the oxyanion hole, in G3.v1 and v3. Mechanisms of drug resistance due to mutations at positions 80 [143,144,145], 155 [67,146], 156 [147], 123 [65,66], and 168 [148,149,150,151] have been well-investigated [160,161], and the importance of these residues in determining the specificity of inhibitors is substantial. RASs R123T and D168Q, in addition to mutation at the catalytic triad residue H57R (G3.v1), RASs R123T and Q80K (G3.v2), and RASs R123T and R155G, along with mutation at catalytic S139P in G3.v3, inhibited interaction of cpd-217 with Gly137. Moreover, R123T and D168Q along with another RAS A156T weakened the association of cpd-217 with G3.v11, resulting in no H-bond formation or hydrophobic interactions (Figure 5A–N).

However, the success of virtual screening is delineated by finding novel chemical structures, and new scaffolds are clearly preferred compared to already known scaffolds. Moreover, due to the ability of the covalent inhibitor to bind irreversibly to off-target proteins, which could lead to toxicological effects, such as immune responses, we utilised the SEA (Similarity Ensemble Approach; http://sea.bkslab.org (accessed on 25 April 2021)) server to identify the presence of similar chemical scaffolds of cpd-217, based on the set-wise chemical similarities against the ChEMBL database. The results indicated no structural similarity with any known anti-viral inhibitors (Supplementary Table S10). We further predicted the unfavourable side-effects due to off-target effects of known molecules and drugs; the SwissTargetPrediction web server (http://www.swisstargetprediction.ch (accessed on 20 March 2022)) was utilised, which combines different measures of chemical similarity based on both chemical structure (2D) and molecular shape (3D). The cpd-217 was found to have less than 0.1 off-target probability based on cross-validation analysis in the ChEMBL database for human protein ligands (Supplementary Table S11).

### 3.5. The Stability and Flexibility of cpd-217 through MD Simulation

**Figure 6:**
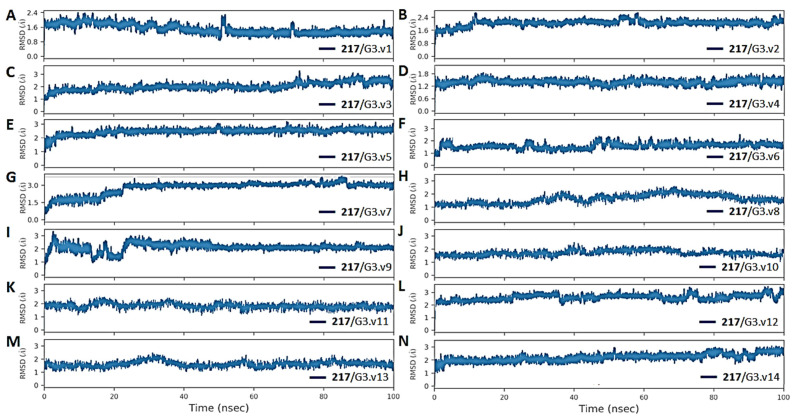
Mean Square Deviation (RMSD) plots of the backbone atoms (CA, N, C) of the HCV NS3/4A G3 variants and heavy atoms of cpd-217 relative to the initial structure over a 100 ns molecular dynamics simulation. The RMSD values are plotted as a function of time (ns) for each variant of compound 217: (**A**) cpd217/G3.v1, (**B**) cpd217/G3.v2, (**C**) cpd217/G3.v3, (**D**) cpd217/G3.v4, (**E**) cpd217/G3.v5, (F) cpd217/G3.v6, (**G**) cpd217/G3.v7, (**H**) cpd217/G3.v8, (**I**) cpd217/G3.v9, (**J**) cpd217/G3.v10, (**K**) cpd217/G3.v11, (**L**) cpd217/G3.v12, (**M**) cpd217/G3.v13, (**N**) cpd217/G3.v14. Each plot illustrates the structural stability and conformational changes of the compound-protease complexes over time, with the RMSD measured in Ångströms (Å).

**Figure 7:**
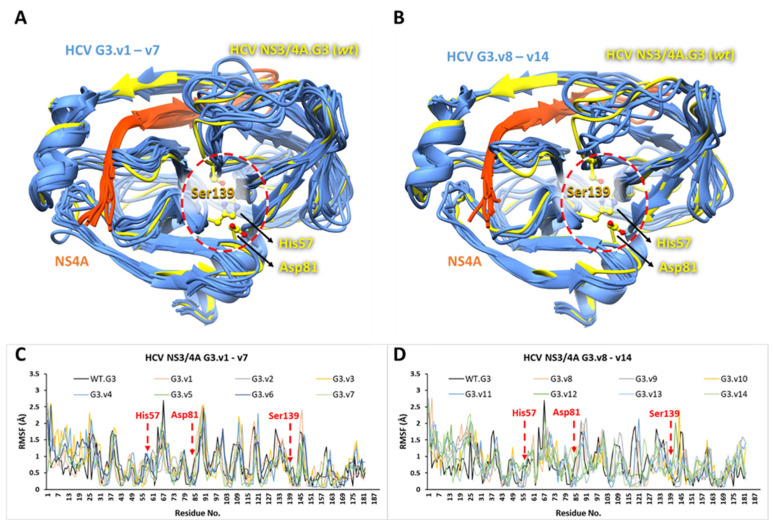
Root mean square deviation of HCV NS3/4A G3 wildtype (WT) with all G3 variants. The MD-simulated HCV NS3/4A G3.WT (yellow) is superimposed on G3 variants (cornflower blue), G3.v1–v7 in (**A**) and G3.v8–v14 in (**B**), after 100 ns. The NS4A is coloured orange red and catalytic triad residues are represented by a ball and stick representation (yellow). The RMSF plots of HCV NS3/4A G3.WT (black line) with G3.v1–v7 in (**C**) and G3.v8–v14 in (**D**) are displayed. The residue numbers are along the x-axis and fluctuations in Å are along y-axis, while catalytic triad residues are highlighted in red.

Molecular dynamics studies have emerged as a reliable method for investigating the stability of protein–ligand complexes [162,163,164,165,166]. Recently, these methods have been combined with more sophisticated binding free energy calculations (e.g., MM/GBSA) to explore the drug resistance mechanisms of HCV resulting from several key mutations of NS3/4A [57,58,167,168]. Here, docking energies expounded only the initial conformation of the cpd-217 at the active site of NS3/4A G3 variants. These complexes were further exploited through MD simulations to predict the most likely binding mode between cpd-217 and the associated residues in all G3 variants (v1 to v14). The present study is primarily involved in the conformation of cpd-217 in G3 variants. Therefore, we were interested in analysing the stability and conformational flexibility of the cpd-217 bound to each G3 variant and corresponding energy contributions of potential residues towards the ligand. To elucidate the dynamic stability and to ascertain the rationality of the ligand sampling, root mean square deviation (RMSD) and root mean square fluctuation (RMSF) values of protein backbone atoms of each G3 variant and heavy atoms of cpd-217 relative to the respective initial structures were calculated, and RMSD/RMSF trajectories were analysed throughout 100 ns. The representative RMSD and RMSF trajectory plots of each G3 variants with bound cpd-217 are displayed in Figure 6A–N and Figure 7A–D.

Overall, the RMSD values of protein backbone atoms (in all variants) and heavy atoms of cpd-217 remained within 3Å throughout the simulation period, except minor variations (slightly higher than 3Å), which were observed in G3.v7 and G3.v9 within the initial 30 ns. The RMSD of backbone atoms of the order of 1–3 Å with no significant conformational change confirmed that the system is well-equilibrated, and cpd-217 remained stable inside of the binding pocket of all NS3/4A variants during the simulation period [169,170,171]. Meanwhile, RMSFs highlighted the flexible regions of G3.WT and G3 variants. No pronounced Cα-RMSF differences occurred, except a few small fluctuations for residues 65 to 72 and 85 to 90. Moreover, no gradual fluctuations were observed in the catalytic site, which evinced the favourable conformation of cpd-217 inside of the binding pocket and depicted convergence of catalytic triad residues (<1 Å) throughout the simulation period.

### 3.6. Binding Free Energy Calculated Using the MM/GBSA Method

**Figure 8:**
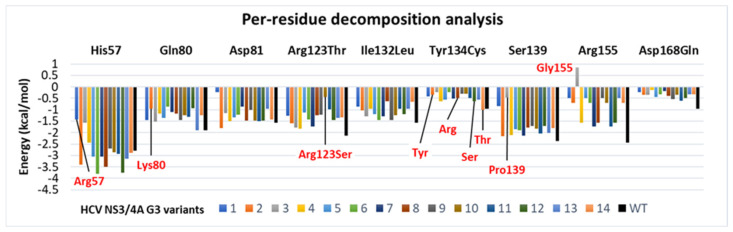
Per-residue decomposition analysis of the potential binding site residues of HCV NS3/4A G3 variants in the presence of cpd-217. The colour codes are represented for each variant, while the wildtype (PDB ID: 4A92) is coloured black. The mutated residues in corresponding variants are highlighted in red. The values are measured in kcal/mol.

The MM/GBSA method has been widely employed in improving the protein–ligand docking results [170,171,172] considering the protein backbone dynamics, electrostatic, van der Waal (vdW), and entropic contributions on the overall binding energy of the complex. The MM/GBSA approach has been widely used in expounding the mechanisms of mutation-induced drug resistance [58,173,174,175,176,177,178,179]. MMGBSA total binding free energy values were extracted from initial frames (avg. of 1000 snapshots from 0.01 to 0.5 ns and referred to as “before MD” or “initial”) and final frames (avg. of 10000 snapshots from last 50 ns, after every 5.0 ps, and referred to as “after MD”). The total binding free energies (ΔGtotal) and energetic components are tabulated in Table 2. We also calculated the affinity score variation (ΔAS) between WT and G3 variants scores to examine the impact of mutation in overall binding with the cpd-217. To further evaluate the binding stability during simulation, energy shift (ΔΔGshift) was incorporated, which supported the stabilising effect of the mutation on ligand binding in protein/ligand complex formation, i.e., a more negative value represents a more favourable stabilising effect in complex with cpd-217. According to the MMGBSA values during MD simulations (Table 2), the overall binding free energy (ΔGbind) of WT was −14.4 kcal/mol. The major energy contributions to the ligand-binding were due to the favourable van der Waals interactions for the WT/cpd-217 complex (ΔEvdw = −41.7 kcal/mol) compared to electrostatic interactions (ΔEele = −32.8 kcal/mol). In contrast, total solvation energies (ΔGsol) showed unfavourable contributions. These values of WT were compared with the binding energies of all G3 variants. The lower binding energy agrees with a higher affinity towards complex stability and vice versa [172,180]. All G3 variants (v1 to v14) possess a few common mutations at Arg123, Ile132, and Asp168, along with other specific mutations, and the quantitative information regarding important residues’ contribution towards cpd-217 binding was exploited through per-residue decomposition analysis (Figure 8). 

In Table 2, the ΔGbind of the WT complex was more favourable than the G3 variants, except G3.v2 and v14 (−15.6, and −15.1 kcal/mol). On the contrary, the G3 variants with lower binding affinities indicated that the specific mutations in these variants will trigger drug resistance to a certain extent; this phenomenon has been explicitly elaborated in several molecular-modelling-assisted drug-resistance studies [53,179,181,182,183,184,185]. Likewise, in WT, it was obvious that the ΔEvdw contribution is the main component in the total binding affinity in each variant (which ranged from −33.5 to −44.2 kcal/mol), and the following was the ΔEele (which ranged from −26.4 to −32.5 kcal/mol). The total solvation energies (ΔGsol) that counteract the electrostatic interactions of all systems were within a difference of <3 kcal/mol from the WT (36.4 kcal/mol). The contributions of the conformational entropy (-TΔS) in all variants ranged between 15.2 and 25.9 kcal/mol. It was observed that the conformational entropic contributions for the complexes have no impact on the order of the ligand’s free energy of binding (ΔGbind). Therefore, the van der Waals contribution was considered more crucial for cpd-217 interaction with NS3/4A protease and differentiating the binding affinities among these variants.

Amongst the G3 mutants, only G3.v2 and G3.v14 displayed a negative affinity variation (ΔAS) of −1.2 and −0.7 kcal/mol, respectively, and they depicted more favourable binding affinity with cpd-217. All other G3 variants displayed a positive affinity variation (ΔAS) as low as +2.8 and as high as +8.1 kcal/mol, and they exhibited lower binding affinity than WT. In particular, the ΔGbind of G3.v4 to G3.v7 and G3.v9 to G3.v11 were approximately the same (ranging from −9.1 to 9.9 kcal/mol, with <−1 kcal/mol variation) and exhibited a similar positive correlation (ΔAS ranged from +4.6 to +5.3 kcal/mol). The trend was likely due to the presence of four combined mutations (R123T + I132L + Y134C + D168Q) in these variants (except G3.v10 with the R123S mutation), which triggered a similar impact on cpd-217 binding, as can be observed from converged protein backbone deviation (<3 Å) (Figure 6). Overall, the variants with R123T + I132L + Y134C + D168Q mutations displayed low binding affinity compared to the WT. More in-depth per-residue decomposition analysis of these variants further supported the underlying impact of these mutations upon ligand binding (Figure 8). Among these G3 variants (G3.v4 to G3.v7, G3.v9 to G3.v11), the interaction energy (ΔGresidue) between cpd-217 and mutated residues R123T (−0.99 to −1.84 kcal/mol, while −0.44 kcal/mol in R123S in G3.v10), I132L (−0.95 to −1.46 kcal/mol), Y134C (−0.23 to −0.63 kcal/mol), and D168Q (−0.14 to −0.61 kcal/mol) were less favourable than those in the WT complex (−2.14, −1.56, −0.96, −0.95 kcal/mol). Moreover, R123T + I132L + Y134C + D168Q mutations in these variants (v4 to v7, v9 to v11) further reduced the energy contributions by Gln80 (−0.8 to −1.45 kcal), Asp81 (−0.87 to −1.51 kcal/mol), and Ser139 (−1.72 to −2.13 kcal/mol) compared to its WT complex (−1.89, −1.56, and −2.36 kcal/mol). 

G3.v3 displayed the highest positive affinity variation (ΔAS = +8.1 kcal/mol) and showed the lowest binding affinity of −6.3 kcal/mol among all G3 variants. The underlying impact of G3.v3 on cpd-217 was mainly due to the S139P mutation and combined effects of R155G and D168Q mutations. Note that the essential function of Asp168 is to stabilise the conformation of Arg155 to maintain the favourable interaction between Arg155 and the ligand [53,54,58,81,161,167,186,187,188]. On the other hand, proline residues are known to cause short- and long-range disruptive changes in secondary structural elements by causing steric hindrance [189]. Ser139 is one of the important catalytic residues, and substitution of this residue has been reported in altered protease activity [190]. An incorporation of glycine at position 155 completely diminished the important interactions with the ligand. At the same time, the substitution of S139P further augmented the impact on cpd-217 binding, as evidenced by the lowest energy shift (ΔΔSshift) of only −2.3 kcal/mol. The contribution energies by these substituted residues further delineated the binding profile with cpd-217. In G3.v3, the interaction energy was abruptly reduced between cpd-217 and mutated residues Pro139 and Gly155 (−0.46 and +0.86 kcal/mol) compared to Ser139 and Arg155 (−2.36 and −2.45 kcal/mol) in the WT complex. Moreover, it also impacted the other catalytic triad residues where the interaction energies (ΔGresidue) of His57 and Asp81 were reduced to −1.56 and −1.14 kcal/mol compared to the WT complex (−2.8 and −1.56 kcal/mol). Hence, the binding affinity of G3.v3 was the lowest compared to WT. Likewise, H57R in G3.v1 exhibited a similar impact on the catalytic triad, where the incorporation of the long side chain of Arg57 obstructed the binding site, which posed the cpd-217 in a different conformation, as can be seen from a sudden fluctuation at ~50 ns in Figure 6A, which remained stable afterwards. The Arg57 displayed less interaction energy (−1.42 kcal/mol) compared to His57 in WT, and the conformation of the side chain of Arg57 further reduced the interaction energy of Asp81 (−0.24 kcal/mol) and Ser139 (−0.84 kcal/mol) compared to the WT complex.

G3.v2 and v14 showed negative affinity variation among all G3 variants and displayed slightly higher binding affinities of −15.6 and 15.1 kcal/mol compared to WT. In G3.v2, the Q80K slightly increased the interaction energy of adjacent Asp81, from −1.56 (WT) to −1.8 kcal/mol, while in G3.v14, the Y134T instead of Y134C (as seen in other G3 variants) along with other mutations triggered a combined effect towards cpd-217 (Table 2). The underlying impact in these two variants (G3.v2 and v14) with bound cpd-217 was evident from the highest energy shift (−8.7 and −9.9 kcal/mol, respectively) during the simulation period, which had a stabilising effect.

To summarise, the structural and pharmacophoric characteristics described here can be utilised to find new leads in compound databases and to design new inhibitors that target G3 variants. This work has supported the idea that further optimisation of structural and pharmacophore properties can lead to developing a multifunctional small-molecule inhibitor that targets all of the common G3-specific mutations. Future efforts would be necessary to create novel multi-functional anti-HCV inhibitors to study the structural and pharmacophore properties of cpd-217 that are responsible for its diverse activity against all G3 variants.

## 4. Conclusions

This work reports the drug-resistant mutations within ligand-binding residues, including the catalytic triad of HCV NS3/4A. The reported mutations are specific for HCV NS3/4A Genotype 3 (G3), which is prevalent in developing countries, including Pakistan. We report mutations within the catalytic triad residues H57R and S139P and RASs Q80K, R123T, R155G, A156T, and D168Q in HCV NS3/4A G3 that hinder the interaction of drugs with Gly137 of the oxyanion hole. This work highlights the pharmacoinformatic approaches utilised to identify a potential covalent inhibitor of HCV NS3/4A G3 to treat Hepatitis C virus. Several ligands and FDA-approved marketed drugs were used to generate a pharmacophore with a similar scaffold to screen multiple small-molecule libraries. Pharmacophore-based virtual screening (PBVS) followed by a covalent docking protocol identified cpd-217 (CHEMBL569970; PubChem45485999) as a potential inhibitor of HCV NS3/4A G3 serine protease. The binding affinity, molecular interactions, and stability of binding of the lead compound were investigated using a molecular docking protocol and MD simulations analysis. The potential warhead identified in this work can serve as a guideline to design covalent inhibitors targeting the catalytic Ser139 considering G3-specific drug-resistant mutations within HCV NS3/4A. The proposed inhibitor may play a key role in expediting the drug discovery process and can be tested in clinical trials to treat HCV. This approach can provide a plethora of energetic information, including the binding free energy between the protein and the ligand, in addition to enriched structural–dynamical information of protein complex structures in solution. Such information is critical for understanding the nature of protein–ligand interactions and guiding drug design and development, which experimental techniques struggle to readily provide.

## Figures and Tables

**Table 1:** Pharmacokinetic and pharmacodynamic properties of CHEMBL569970 (cpd-217; PubChem45485999) along with post-docking analysis and binding interactions with HCV NS3/4A G3 variants (v1 to v14).

Molecule Name	RMSD	Drug-likeness	Mutagenic	Tumorigenic	cLogP	cLogS	Polar Surface Area	Reproductive Effective	Irritant
cpd-217	0.121	−0.402	none	none	−0.488	−1.886	129.16	none	none
Docking Score and Binding Interactions of cpd-217									
HCV NS3/4A G3 Variant No.	Docking Score (kcal/mol)	Mutations Within NS3/4A G3 Sequences	Ligand-Binding Residues	Covalent Bond With Ser139	No. of H-Bonds	H-Bond (Residues Involved)	Salt Bridge (Residues Involved)	Pi–Cation Interaction (Residues Involved)	Pi–Pi Interaction (Residues Involved)
1	−6.588	His57Arg, Arg123Thr, Ile132Leu, Tyr134Cys, Asp168Gln	Gln41, Thr42, Phe43, Val55, Arg57, Gly58, Leu132, Leu135, Lys136, Ser138, Ser139, Phe154, Arg155, Ala156, Ala157	Yes	5	Gln41, Thr42, Arg57, Leu135, Arg155	-	-	-
2	−5.308	Gln80Lys, Arg123Thr, Ile132Leu, Asp168Gln	Gln41, Phe43, His57, Gly58, Leu132, Leu135, Lys136, Ser138, Ser139, Phe154, Arg155, Ala156, Ala157	Yes	4	Gln41, His57, Leu135, Gly137	-	-	-
3	−5.447	Arg123Thr, Ile132Leu, Tyr134Cys, Ser139Pro, Arg155Gly, Asp168Gln	His57, Val78, Asp79, Asp81, Ala156, Met485, Phe486, Asp487, Ser488, Val524, Cys525, Gln526, His528	No (S139P)	3	Asp81, Asp487, Gln526	Asp81	-	-
4	−6.397	Arg123Thr, Ile132Leu, Tyr134Cys, Asp168Gln, Cys525Trp	Gln41, Phe43, Val55, His57, Gly58, Leu132, Leu135, Lys136, Gly137, Ser138, Ser139, Arg155, Ala156, Ala157, Val158, Cys159, Gln526, His528	Yes	4	Gly137, Ser139, Ala157	-	-	His57
5	−5.986	Arg123Thr, Ile132Leu, Try134Cys, Asp168Gln, Cys525Tyr	Gln41, Val55, His57, Leu132, Leu135, Lys136, Gly137, Ser138, Ser139, Phe154, Arg155, Ala156, Ala157, Val158, Cys159, Gln168	Yes	4	Gly137, Ser139, Ala157	-	-	-
6	−5.101	Phe43Leu, Arg123Thr, Ile132Leu, Tyr134Cys, Asp168Gln	Gln41, Thr42, Leu43, His57, Gly58, Leu132, Lys136, Gly137, Ser138, Ser139, Phe154, Arg155, Ala156, Ala157, Cys159, Gln526, His528	Yes	3	Thr42, Gly137, Ala157	-	-	-
7	−5.395	Arg123Thr, Ile132Leu, Tyr134Cys, Asp168Gln, Gln526His	His57, Leu132, Ala133, Leu135, Lys136, Gly137, Ser138, Ser139, Phe154, Arg155, Ala156, Ala157, Met485	Yes	2	Leu135, Gly137	-	-	-
8	−5.630	Arg123Thr, Ile132Leu, Tyr134Arg, Asp168Gln	Gln41, His57, Gly58, Leu132, Leu135, Lys136, Gly137, Ser138, Ser139, Phe154, Arg155, Ala156, Ala157, Cys159, Gln526	Yes	3	His57, Leu135, Gly137	-	-	-
9	−5.184	Arg123Thr, Ile132Leu, Tyr134Cys, Val158Ala, Asp168Gln	Gln41, His57, Gly58, Leu132, Leu135, Lys136, Gly137, Ser138, Ser139, Phe154, Arg155, Ala156, Ala157, Cys159, Gln526	Yes	3	Gln41, His57, Arg155	-	Lys136	-
10	−4.722	Arg123Ser, Ile132Leu, Tyr134Cys, Asp168Gln	Gln41, His57, Gly58, Leu132, Leu135, Lys136, Gly137, Ser138, Ser139, Phe154, Ala156, Ala157	Yes	4	His57, Leu135, Ser139	-	-	-
11	−4.169	Arg123Thr, Ile132Leu, Tyr134Cys, Ala156Thr, Asp168Gln	Gln41, His57, Leu132, Leu135, Lys136, Gly137, Ser138, Ser139, Phe154, Arg155, Thr156, Ala157, Met485, Gln526	Yes	0	-	-	-	-
12	−5.288	Arg123Thr, Ile132Leu, Tyr134Ser, Asp168Gln	Gln41, His57, Gly58, Leu132, Leu135, Lys136, Gly137, Ser138, Ser139, Phe154, Arg155, Ala156, Ala157, Met485	Yes	6	Gln41, His57, Leu135, Ser139	-	-	-
13	−5.392	Arg123Thr, Ile132Leu, Tyr134Cys, Asp168Gln, Gln526Arg	Gln41, Phe43, Val55, His57, Asp81, Leu132, Leu135, Lys136, Gly137, Ser138, Ser139, Phe154, Arg155, Ala156, Ala157, Met485, Arg526	Yes	2	Asp81, Gly137	Asp81	-	-
14	−6.153	Arg123Thr, Ile132Leu, Tyr134Thr, Cys159Val, Asp168Gln	Gln41, Thr42, Phe43, His57, Gly58, Leu132, Leu135, Lys136, Gly137, Ser138, Ser139, Phe154, Arg155, Ala156, Ala157, Val159	Yes	5	Gln41, Gly137, Ser139, Ala157	-	-	-

**Table 2:** Predicted MMGBSA calculations throughout 100 ns for HCV NS3/4A G3 variants along with it WT complexed with cpd-217.

HCV	MMGBSA Energy Components (kcal/mol)							Energy Shift ΔΔGshift ^a^	Variation (ΔAS) ^b^
	Before MD		After MD (Last 50 ns)						
	ΔGtol	ΔEele	ΔEvdw	ΔEMM	ΔGsol	-TΔS	ΔGbind		
G3.v1	−5.7	−32.5	−44.2	−76.7	39.1	25.9	−11.6	−5.9	2.8
G3.v2	−6.9	−29.1	−43.2	−72.3	36.4	20.3	−15.6	−8.7	−1.2
G3.v3	−4.0	−27.3	−37.1	−64.4	35.4	22.7	−6.3	−2.3	8.1
G3.v4	−4.4	−32.4	−38.1	−70.5	38.2	22.4	−9.9	−5.5	4.5
G3.v5	−3.8	−31.1	−42.3	−73.4	39.5	24.7	−9.2	−5.5	5.1
G3.v6	−4.1	−29.1	−40.2	−69.3	37.2	23.0	−9.1	−5.0	5.3
G3.v7	−3.8	−26.4	−36.6	−63.0	38.2	15.2	−9.6	−5.8	4.8
G3.v8	−3.8	−31.0	−36.4	−67.4	37.2	19.0	−11.2	−7.4	3.2
G3.v9	−3.1	−27.4	−33.5	−60.9	36.4	15.3	−9.2	−6.2	5.2
G3.v10	−3.0	−28.1	−37.1	−65.2	36.1	20.0	−9.1	−6.1	5.3
G3.v11	−4.7	−30.5	−35.5	−66.0	38.2	18.0	−9.8	−5.1	4.6
G3.v12	−4.4	−31.4	−40.1	−71.5	39.1	21.0	−11.4	−7.0	3.0
G3.v13	−5.0	−27.4	−39.2	−66.6	37.6	18.5	−10.5	−5.5	3.9
G3.v14	−5.2	−32.1	−43.3	−75.4	38.2	22.1	−15.1	−9.9	−0.7
**WT**	−5.7	−32.8	−41.7	−74.5	36.4	23.7	−14.4	−8.6	0.0

Note: ^a^ ΔΔGbind represents the energy shift, which a complex attained throughout 100 ns. A lower value indicates a higher binding affinity and favourable stabilising impact over the simulation period. ^b^ ΔAS represents affinity score variation. For binding energy, lower values represent more affinity with the protein.

## Data Availability

The sequences were retrieved from NCBI (https://www.ncbi.nlm.nih.gov/ (accessed on 25 April 2021)) and structures were downloaded from PDB (https://www.rcsb.org/ (accessed on 25 April 2021)), which are freely available. For the in silico analyses, several online resources were utilised, which are available free for use. Sequences were aligned using Clustal Omega (https://www.ebi.ac.uk/Tools/msa/clustalo/ (accessed on 15 April 2021)), while the template for homology modelling was identified using BLAST (https://blast.ncbi.nlm.nih.gov/Blast.cgi (accessed on 15 April 2021)). For alignment visualisation, ESPript 3.0 (https://espript.ibcp.fr/ESPript/ESPript/ (accessed on 20 March 2022)) was used. Homology modelling was performed using SWISS-MODEL (https://swissmodel.expasy.org/ (accessed on 25 April 2021)). For structural refinement and assessment, web servers, including GalaxyRefine (http://galaxy.seoklab.org/cgi-bin/submit.cgi?type=REFINE (accessed on 25 April 2021)), FATCAT (https://fatcat.godziklab.org/ (accessed on 25 April 2021)), ERRAT (https://servicesn.mbi.ucla.edu/ERRAT/ (accessed on 28 April 2021)), ProSA (https://prosa.services.came.sbg.ac.at/prosa.php (accessed on 28 April 2021)), and MolProbity (http://molprobity.biochem.duke.edu/ (accessed on accessed on 8 May 2022)) were used, while Verify 3D and PROCHECK were accessed from https://saves.mbi.ucla.edu/ (accessed on 15 May 2022). Binding site residues were identified using freely downloadable software, including LigPlot (https://www.ebi.ac.uk/thornton-srv/software/LigPlus/download.html (accessed on 20 April 2021)), and Chimera (https://www.cgl.ucsf.edu/chimera/download.html (accessed on 10 April 2024)) was used for the active site analysis. Small molecule database screening was performed using the Pharmit online resource (https://pharmit.csb.pitt.edu/search.html (accessed on 12 June 2021)). For pharmacophore design, covalent and molecular docking studies, and ligand interaction analysis, Maestro, Schrodinger was used, which offers a trial learner’s license for use (https://www.schrodinger.com/products/maestro (accessed on 23 September 2021)). Molecular dynamics simulations and free energy calculations were performed using the AMBERTools Suite (https://ambermd.org/index.php (accessed on 4 February 2022)).

## Supplementary Materials

The following supporting information can be downloaded at: https://www.mdpi.com/article/10.3390/v16081250/s1. Supplementary Figure S1: Structural representation of HCV NS34A. Supplementary Figure S2: Ramachandran Analysis of HCV NS34A G3 Variants. Supplementary Figure S3: ROC Analysis of AAHH pharmacophore hypothesis. Supplementary Figure S4: The best pharmacophore model AAHH. Supplementary Figure S5: Two-dimensional (2D) chemical structures of compounds obtained using covalent docking. Supplementary Figure S6: Compounds obtained using glide docking-based virtual screening. Supplementary Figure S7: Interaction analysis of alpha-ketoamide inhibitor Boceprevir (PDB2OC8). Supplementary Table S1: HCV NS34A Binding Site Analysis. Supplementary Table S2: Ligand-binding residues of HCV NS34A. Supplementary Table S3: Representative Sequences for Identified Mutations. Supplementary Table S4: Template identification for modeling HCV NS34A G3 variants using BLAST. Supplementary Table S5: Structural Evaluation of HCV NS34A G3 Variant Models. Supplementary Table S6: List of Inhibitors reported for HCV NS34A. Supplementary Table S7: List of ligands filtered for docking. Supplementary Table S8: List of compounds obtained using covalent docking. Supplementary Table S9: Compounds obtained using glide docking-based virtual screening. Supplementary Table S10: SEA results. Supplementary Table S11: SwissTargetPrediction.
