# Supplementary figures and images for: Pharmacophore-Assisted Covalent Docking Identifies a Potential Covalent Inhibitor for Drug-Resistant Genotype 3 Variants of Hepatitis C Viral NS3/4A Serine Protease
Source: Viruses. 2024 Aug 3;16(8):1250. doi: 10.3390/v16081250 (PMC11359326; doi:10.3390/v16081250)

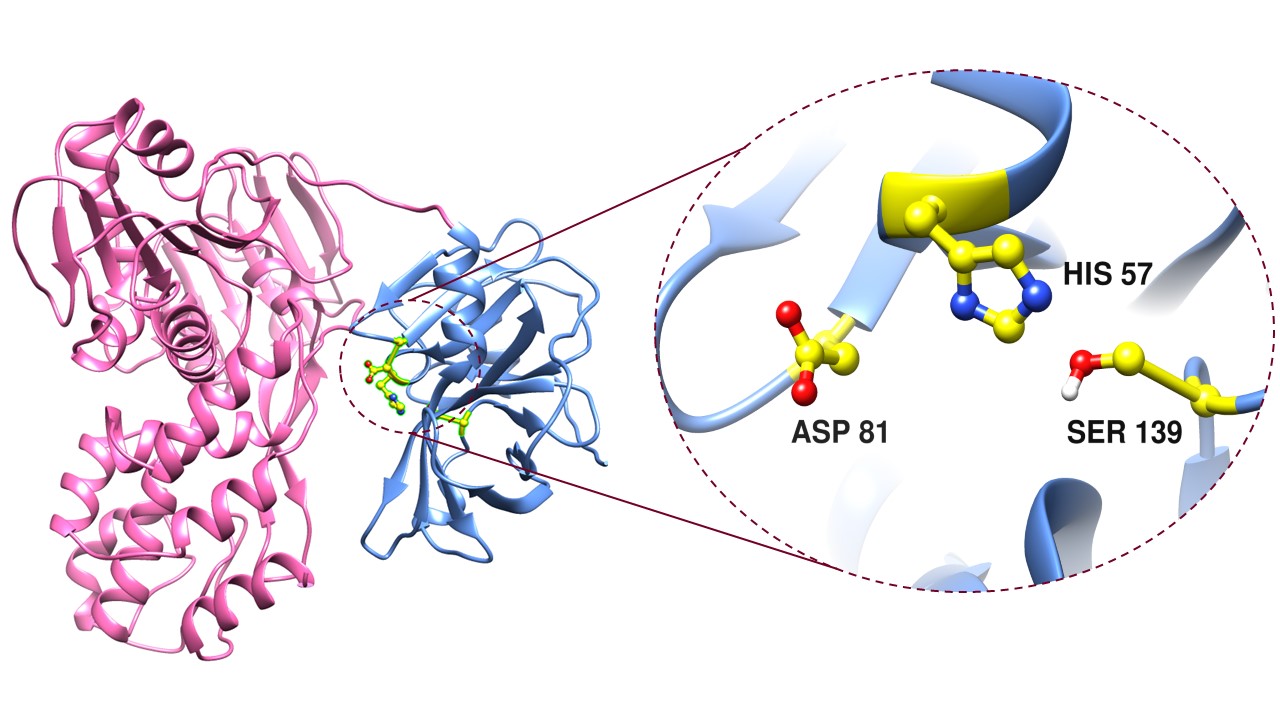

Supplement: Supplementary file 1 [file viruses-16-01250-s001.zip › Supplementary Figure S1 Structural representation of HCV NS34A.jpg]

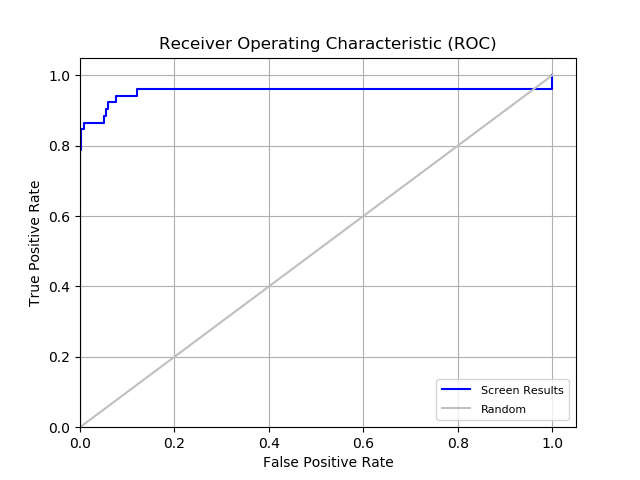

Supplement: Supplementary file 1 [file viruses-16-01250-s001.zip › Supplementary Figure S3 - ROC Analysis of AAHH pharmacophore hypothesis.png]

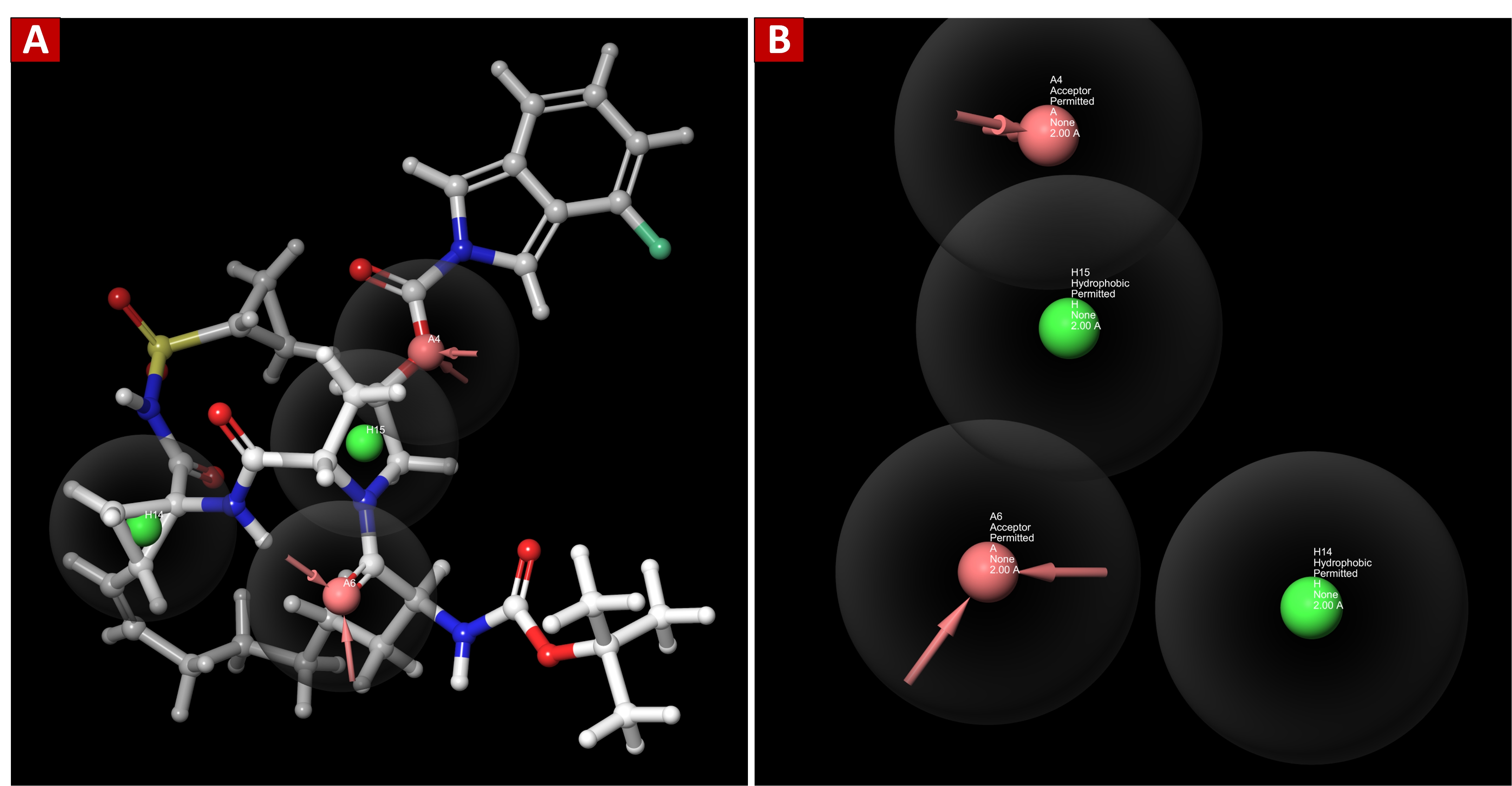

Supplement: Supplementary file 1 [file viruses-16-01250-s001.zip › Supplementary Figure S4 - The best pharmacophore model AAHH.jpg]

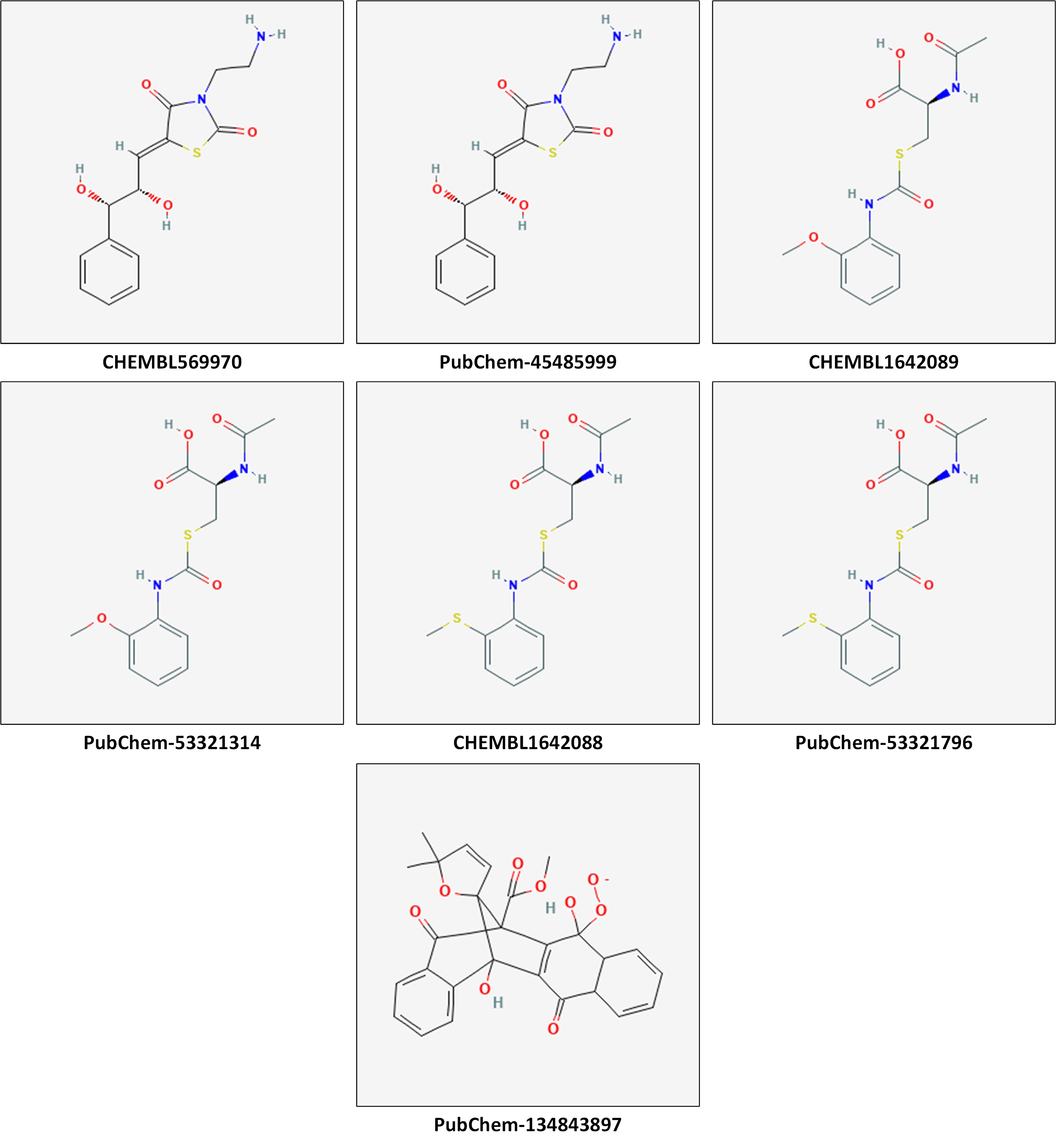

Supplement: Supplementary file 1 [file viruses-16-01250-s001.zip › Supplementary Figure S5 - Two-dimensional (2D) chemical structures of compounds obtained using covalent docking.jpg]

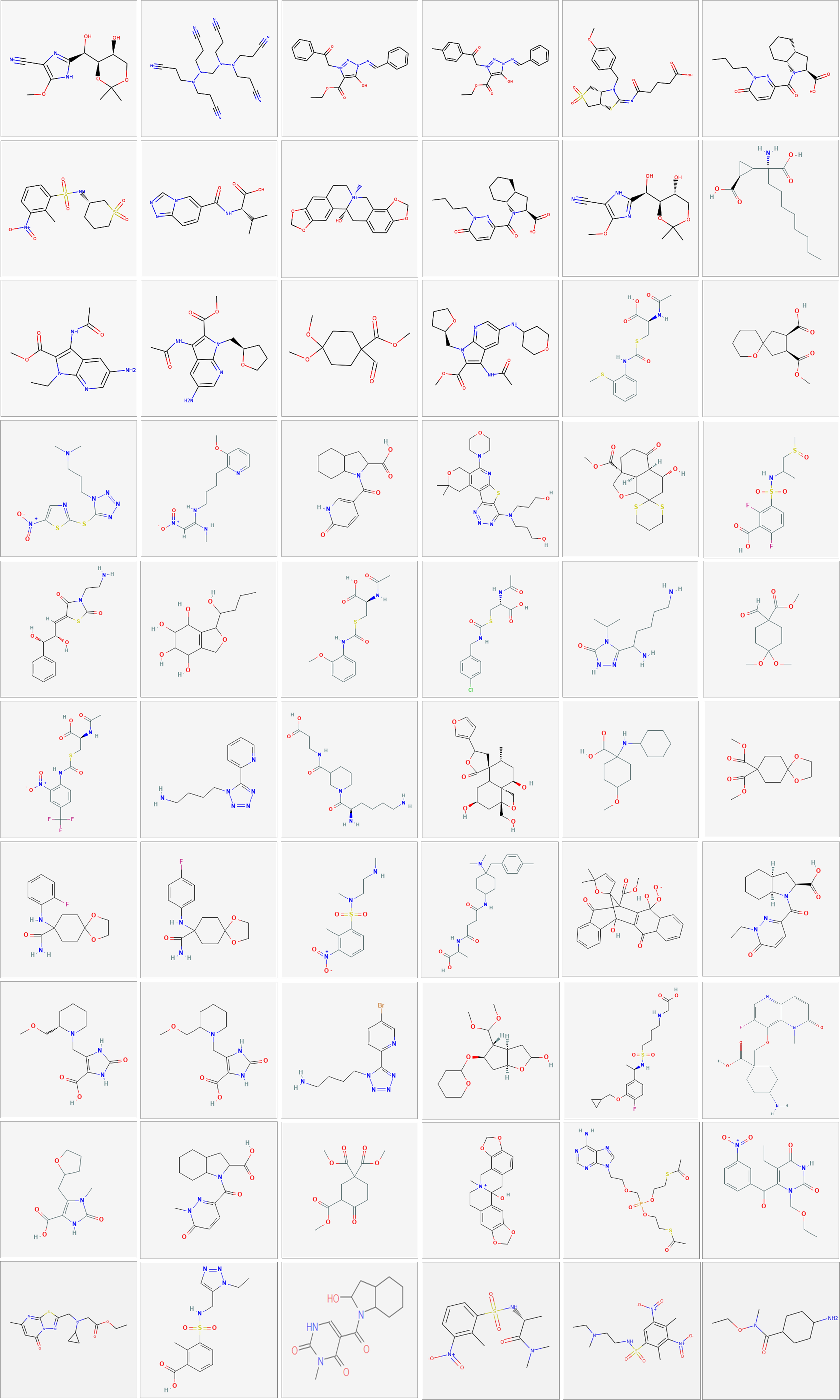

Supplement: Supplementary file 1 [file viruses-16-01250-s001.zip › Supplementary Figure S6 - Compounds obtained using glide docking-based virtual screening.jpg]

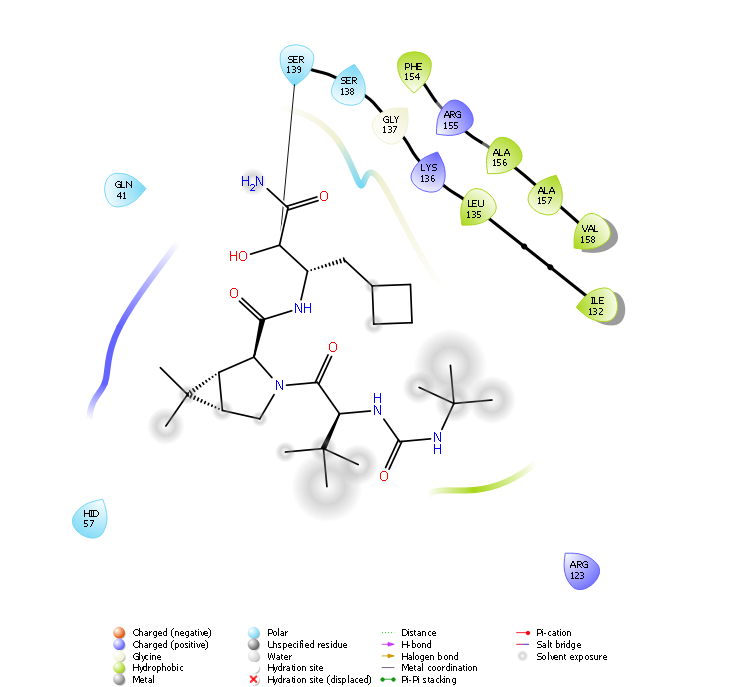

Supplement: Supplementary file 1 [file viruses-16-01250-s001.zip › Supplementary Figure S7 - Interaction analysis of alpha-ketoamide inhibitor Boceprevir (PDB2OC8).png]
